# Proteomic Comparison of MCF-7 Tumoursphere and Monolayer Cultures

**DOI:** 10.1371/journal.pone.0052692

**Published:** 2012-12-20

**Authors:** Brian J. Morrison, Marcus L. Hastie, Yadveer S. Grewal, Zara C. Bruce, Chris Schmidt, Brent A. Reynolds, Jeffrey J. Gorman, J. Alejandro Lopez

**Affiliations:** 1 School of Biomolecular and Physical Sciences, Griffith University, Brisbane, Queensland, Australia; 2 Queensland Institute of Medical Research, Brisbane, Queensland, Australia; 3 McKnight Brain Institute, University of Florida, Gainesville, Florida, United States of America; Shantou University Medical College, China

## Abstract

Breast cancer is a heterogenous disease, composed of tumour cells with differing gene expressions and phenotypes. Very few antigens have been identified and a better understanding of tumour initiating-cells as targets for therapy is critically needed. Recently, a rare subpopulation of cells within tumours has been described with the ability to: (i) initiate and sustain tumour growth; (ii) resist traditional therapies and allow for secondary tumour dissemination; and (iii) display some of the characteristics of stem cells such as self-renewal. These cells are termed tumour-initiating cells or cancer stem cells, or alternatively, in the case of breast cancer, breast cancer stem cells. Previous studies have demonstrated that breast cancer stem cells can be enriched for in “tumoursphere” culture. Proteomics represents a novel way to investigate protein expression between cells. We hypothesise that characterisation of the proteome of the breast cancer line MCF-7 tumourspheres compared to adherent/differentiated cells identifies proteins of novel interest for further isolating or targeting breast cancer stem cells. We present evidence that: (i) the proteome of adherent cells is different to the proteome of cells grown in sphere medium from either early passage (passage 2) or late passage (passage 5) spheres; (ii) that spheres are enriched in expression of a variety of tumour-relevant proteins (including MUC1 and Galectin-3); and (iii) that targeting of one of these identified proteins (galectin-3) using an inhibitor (N-acetyllactosamine) decreases sphere formation/self-renewal of MCF-7 cancer stem cells *in vitro* and tumourigenicity *in vivo*. Hence, proteomic analysis of tumourspheres may find use in identifying novel targets for future therapy. The therapeutic targeting of breast cancer stem cells, a highly clinically relevant sub-population of tumour cells, has the potential to eliminate residual disease and may become an important component of a multi-modality treatment of cancer.

## Introduction

Breast cancer accounts for 18% of all female cancers [1]. As such, development of models to investigate breast cancer for treatment or prevention is a major focus of scientific research. Prominent in the breast cancer field has been the notion of the existence of a transformed population of cells with many of the properties of stem cells (self-renewal, differentiation, long-term culture capacity) that may be responsible for the heterogeneity, origin and maintenance of tumours [2], [3]. These stem cell-like cells designated as breast cancer stem cells (BCSCs) or alternatively as tumour-initiating cells, represent a minor subset of cells in the tumour and are distinct from more differentiated cells. One method of studying BCSCs *in vitro* is based upon work identifying neural stem cells through a cell culture method known as the neurosphere assay, which makes use of serum-free medium supplemented with epidermal growth factor and basic fibroblast growth factor [4], [5]. Application of the neurosphere assay culture conditions have been used to identify undifferentiated human mammary stem cell grown in culture [6] known as “mammospheres” and to identify candidate human BCSCs in breast cancer cell lines [7] known as “tumourspheres.” Sphere culture systems have shown that tumourspheres cultured from human breast cancer cell lines exhibit stem cell-like functional properties such as symmetric division and self-renewal [8] and a variety of phenotypic properties, such as HER2 [9], [10], CD49f [11], protein phosphatase and tensin homolog – PTEN [12], EpCAM [13], [14], mucin 1( MUC1) [15], CD44^+^/CD24^−/low^ populations [13], [14], and aldehyde dehydrogenase 1 – ALDH1 [16], [17] amongst others. Additional candidate stem cell markers are yet to be identified. The widely used MCF-7 breast cancer cell line is a useful model to investigate potential BCSC markers. Whole MCF-7 spheres as well as subpopulations within spheres have been shown to be more tumourigenic than adherent/monolayer parental cultures [7], [11], [18] hinting to an enriched population of BCSC. The proteome of MCF-7 tumourspheres has yet to be defined.

The proteome of a group of cells grown under the same conditions can be defined as the combined set of proteins being expressed by the genomes of those cells at a particular time [19]. Proteomics is the large-scale high-throughput application of proteome research (reviewed in [20]). The study of proteomes from cells can be used to compare two or more groups of cells to identify differences between them. Application of proteomics to the investigation of cancer stem cell models has the potential to identify differences in cell signalling pathways and cell surface phenotype between cancer stem cells and non-cancer stem cells. Identification of cell surface phenotypes is particularly important as this can be used to further isolate cancer stem cells for additional research or as a target of therapy. Proteomics can also compliment other approaches of investigation such as flow cytometry analysis. Proteomic approaches to investigating breast cancer have been performed using both patient samples and cell culture lines, and this has led to the identification of several markers and signalling pathways involved in disease (reviewed in [21]).

Proteome comparisons between MCF-7 cells grown as adherent cells and as spheres and between early and late passage spheres were undertaken in order to investigate the changes occurring in these populations. We hypothesise that proteins that are upregulated on/within spheres compared to adherent cells might be useful for further isolating subpopulations of cells that may be enriched for the properties of cancer stem cells. This approach has identified several candidate proteins that are expressed within spheres, including proteins with known cancer associations. One protein identified as overexpressed within spheres compared to adherent cells, MUC1, was further assessed for cell surface expression using flow cytometry techniques. Another protein identified, galectin-3, was further characterised for expression within adherent cells and tumourspheres using quantitative real-time (RT)-PCR. The use of a ligand (N-acetyllactosamine (LacNAc)) against galectin-3 was also investigated for ability to disrupt sphere formation, a method to assess stem cell self-renewal. This study was conducted to demonstrate the utility of a proteome approach in identifying candidate BCSC markers. Galectin-3 was considered a candidate protein of interest because of its higher expression in spheres compared to adherent cells, its known roles in cancer progression, its expression on the plasma membrane and its ability to be blocked with small molecules.

## Materials and Methods

### Cell Culture Conditions

MCF-7 (ATCC, Rockville, MD, USA) human cells were cultured as adherent cells using RPMI-1640 (Gibco, Invitrogen Australia Pty Limited, Mount Waverley, VIC, Australia) supplemented with 10% foetal calf serum (Hyclone, Logan, UT, USA) and 1% penicillin G-streptomycin solution (Gibco). MCF-7 were grown as tumourspheres by adapting the growth medium and methods used to culture neural stem cells in the neurosphere assay (NSA) [4]. All cells were grown at 37°C in 5% CO_2_. NSA media consists of Dulbecco's Modified Eagle Medium: Nutrient Mixture F-12 (DMEM/F12) (Gibco) containing 20 ng/mL rhEGF (R&D Systems, Minneapolis, MN, USA), 10 ng/mL rhbFGF (R&D Systems), 4 μg/mL heparin (Sigma, St. Louis, MO, USA), 10% human proliferation supplement (NeuroCult®, Stem Cell Technologies Inc., Vancouver, BC, Canada), 0.15% bovine serum albumin (BSA) (Sigma), and 1% penicillin G-streptomycin solution. Spheres are routinely spun at a low speed during centrifugation, typically at 129*g* for five minutes (Eppendorf AG, 5810R centrifuge, Hamburg, Germany). Spheres are disassociated and passaged using 0.05% trypsin-EDTA (Gibco) for two minutes at 37°C. Cells are then pipetted to break up spheres further into single cells with the addition of equal volume trypsin inhibitor. Trypsin inhibitor: 10 mg recombinant bovine DNase 1 (Sigma), 0.14 g trypsin inhibitor from *Glycine max* (soybean) (Sigma), 16 mL HEPES (1 M, Sigma), made up to 1 L MEM (Gibco), then filtered through a 22 μm filter (Millipore, Billerica, USA), aliquoted into 10 mL aliquots and stored at −20°C. Sphere assays were conducted using 1000 cells over passages 1, 2, 4, 6, and 8. Alternatively at passage 6 either 3000, 1000, 300, 100, 30, 10, 3, or 1 cells were plated. Cells were plated in 50 μL of NSA media in the wells of a 384 well optical bottom plate (Nunc Thermo Fisher Scientific, Rochester, NY, USA). Ten wells were used as replicates. After 5 days of culture the number of 3D floating multicellular spheroid clusters (not clumps of cells) formed was counted. Sphere forming efficiency  =  (# of spheres formed/# of single cells plated) x 100. Growth curves for cells grown under adherent conditions and tumoursphere conditions were conducted using T-25 culture flasks and with 250,000 single cells for the start of culture in triplicate. At day 5 adherent cells were 75–95% confluent. Cells were passaged and counted every 5 days and cultured again at the starting frequency. Experiment repeated multiple times, representative results shown.

### Proteome Determination

General biochemical reagents were obtained from Sigma–Aldrich. Acids and organic solvents were HPLC grade or better. Offgel reagents including; 13 cm pH3-10 IPG DryStrips, IPG strip covering fluid, pH3-10 IPG buffer, kits for protein enrichment (2-D Clean-up kit) and quantitation (2-D Quant kit) were obtained from GE Healthcare (Uppsala, Sweden). Trypsin (modified sequencing grade, bovine pancreas) was purchased from Roche Diagnostics. Water was purified using a Milli-Q Synthesis system (Millipore). Samples used for comparing the proteome include 3 matched biological replicates of passage 2 and 5 MCF-7 tumourspheres and adherent cells. Tumourspheres and adherent cells are prepared as previously described. Sub-confluent adherent cells were washed two times with PBS (30–35 mL) and then scraped off in a small amount of PBS using a sterile cell scraper. Samples were then washed an additional time in 30–35 mL PBS and transferred to 1.5 mL cryotubes (Nunc). Tumourspheres were harvested and washed three times with PBS (no trypsin treatment) before being transferred to 1.5 mL cryotubes. Cells were then snap frozen using a slurry of dry ice and absolute ethanol and stored at −80°C until use. Cells were lysed in lysis buffer: 7 M urea, 2 M Thiourea, 4% CHAPS 50 mM Tris-HCl pH 8.8 containing 1x phosphtase inhibitors (Sigma phosphatase inhibitor cocktail II, sodium molybdate, sodium orthovanadate, sodium tartrate and imidazole).

#### Protein Enrichment, Quantitation

Lysates were individually enriched for proteins using a 2-D Clean-Up Kit (GE Healthcare) and protein quantified using the 2D Quant Kit (GE Healthcare) as described previously [22].

#### Protein tryptic digestion

To 300 µg of protein enriched cell lysate SDS was added to 1% and proteins reduced by the addition of DTT to 10 mM (2 hours at 4°C, 2 hours at 22°C). Proteins were then alkylated by the addition of iodoacetamide to 25 mM (1 hour at 22°C in the dark). Three micrograms of trypsin was coprecipitated with the reduced and alkylated protein by the addition of 9 volumes of methanol at −20°C (16 hours at −20°C). Protein precipitates were collected by centrifugation at 4°C 20,000×*g* for 5 minutes. Pellets were washed with 1 mL of 90% (v/v) methanol at −20°C. The final pellets were resuspended in 100 µL of 10% (v/v) acetonitrile, 100 mM triethyl ammonium bicarbonate, pH 8.5 and incubated at 37°C for 2 hours followed by the addition of a further 6 µg of trypsin and 6 hours digestion at 37°C. Digests were dried down to 10 μL, a further 100 µL of 10% ACN added then dried down until about 20 µL remained.

#### Peptide In-solution IEF Fractionation

The Offgel procedure was performed as per the manufactures instructions with modifications as noted below. Offgel buffer 1.8 ml (2.4% glycerol, 0.5% 3–10 IPG buffer (GE Healthcare)) was added to each sample. Aliquots (150 µL) of the relevant sample were dispensed into the wells of 12 well OFFGEL sample frames that had been assembled over a 13 cm pH3-10 IPG DryStrip (GE Healthcare) in a tray of the Agilent Technologies 3100 OFFGEL Fractionator according to the accompanying instrument instructions. The samples were focused with a maximum current of 50 μA until 20 kVh was reached using the standard 3100 OFFGEL peptide focusing program (4500 V max voltage). Individual wells were subsequently harvested weighed and made up to 250 mg with 1% formic acid. These were stored at −80°C until required.

#### Capillary(Cap)HPLC-LTQ-Orbitrap

Acidified Offgel fractions were subjected to CapHPLC-MS/MS analysis using an Prominence nano HPLC system (Shiumadzu, Kyoto, Japan) interfaced with a linear ion-trap (LTQ) – Orbitrap XL hybrid mass spectrometer (Thermo Fischer Scientific, Bremen, Germany). Aliquots (30 µL) of the Offgel fractions were loaded onto a 300 Å, 300 μm ×5 mm C18 trap column (Dionex Acclaim® PepMap™ μ-Precolumn) at 30 µL/min in 100% solvent A (0.1% (v/v) aqueous formic acid) for 3.5 minutes at 40^o^C and subsequently back flushed onto a pre-equilibrated analytical column (Vydac Everest C18 300 Å, 150 μm ×150 mm, Alltech) using a flow rate of 1 µL/minute and 98% solvent A, 2% solvent B (80% (v/v) ACN/20% (v/v) H_2_O, containing 0.1% (v/v) formic acid). Peptides were separated and analysed on an LTQ-Orbitrap XL mass spectrometer as described previously [22].

#### Data Processing

Tandem mass spectra were processed using MaxQuant (1.2.2.5) [23]. RAW files were searched using Andromeda against the Uniprot reference human proteome downloaded on 20120323 (canonical and isoform sequence data, 81213 entries). The search included contaminants, fixed modification: carbamidomethyl-cysteine, variable modifications: deamidation (asparagine, glutamine); oxidation (methionine); acetyl (protein N-terminus); enzyme trypsin/P, MS tolerance 20ppm, MSMS tolerance 0.5 Da. Protein, peptide and site FDR 0.01 (apply site FDR separately); minimum peptide length 6; use razor and unique peptides; label free quantitation minimum ratio count 2; use second peptides; requantify; do not use low scoring versions of identified peptides. The results of the search and quantification were then analysed using Perseus (1.2.0.17) [23]. The data was initially filtered to remove proteins “only identified by site”, “reverse” sequence and “contaminant”. Then the log(ln) of the label free quantitation (LFQ) values were taken (filtered 3 valid values per row), an imputed value given to remaining non-existent values (width 0.3, downshift 5); one-way ANOVA and *t*-tests were performed using a p-value of 0.05 and significance was determined using FDR correction (Benjamini-Hochberg). The results from the proteomic analysis were further analyzed through the use of Ingenuity Pathways Analysis (Ingenuity® Systems, www.ingenuity.com).

### Flow Cytometry Analysis

The expression of MUC1 was conducted using fluorescent conjugated antibodies for MUC1-FITC (clone HMPV, Becton Dickinson, BD, San José, CA, USA). The FITC-mouse IgG1, κ isotype control (clone MOPC-21, Biolegend San Diego, California, USA) was used to indicate background staining in all MUC1 experiments. Cells were washed twice in PBS supplemented with 1% BSA (Sigma) before staining with antibodies. The dilutions for all antibodies were 1∶100. Following addition of antibodies, cells were incubated for 20 minutes at 4°C in the dark and washed to remove any unbound antibody. Between 10,000 and 100,000 total events were examined using a FACSCanto II (BD) with data analyzed using FACSDiva (BD) software. Live cells were gated using either propidium iodide (50 µg/mL final concentration, Sigma) or SYTOX Blue (75 µg/mL final concentration, Molecular Probes, Eugene, Oregon, USA) staining. Live-gated cell data was analyzed using FlowJo software (Tree Star, Inc., Ashland, OR, USA) to obtain information such as Δ-median fluorescence intensity (FI) or positivity (%) of MUC1 above the baseline value for the unstained or isotype control. In this case, Δ refers to the parameter in question (median or positivity) with the background staining values removed. Fluorescent markers were measured using either linear or log scales as was deemed appropriate for the expression intensity of the markers measured. Importantly, each technique gives equivalent results in comparison to matched samples measured on the same scale. Experiment repeated multiple times, representative results shown.

### Test of N-acetyllactosamine (LacNAc) Effect on Tumourspheres and Tumourigenicity

Phenotype and sphere forming efficiency of MCF-7 tumourspheres was tested in the presence of LacNAc (Sigma). LacNAc concentrations ranged from 0.1 mM to 6 mM. These experiments were conducted in 384 well plates with 5000 or 1000 single cells plated per well, n = 10. For tumourigenicity studies, six-eight week old NOD.Cg-*Rag1^tm1Mom^Il2rg^tm1Wjl^*/SzJ mice (The Jackson Laboratory, Bar Harbor, Maine, USA) were used for the subcutaneous injection of 2×10^6^ single viable cells derived from passage 2 spheres either cultured in 3 mM LacNac for 6 days or left untreated. Cells were counted and viability assessed using a Countess™ (Invitrogen) automated cell counter. Before transplantation, the mice received 60-day release 17β-estradiol pellets (Innovative Research of America, Sarasota, Florida, USA) placed subcutaneously in the interscapular region prior to cell injection. Single cells were resuspended in 100 µL of 1∶1 matrigel (BD) PBS. Mice (n = 4) were followed every 1–3 days for assessment of tumour size. Volume of the tumours was measured using the formula: (½ × length) × width^2^. The endpoint of the experiments was determined when the tumour volume reached 520 mm^3^. All animal research complied with the local animal ethics committee (Queensland Institute of Medical Research – P1159). All efforts were made to minimize suffering.

### Quantitative Real-time PCR for Galectin-3 Expression

Passage 2 spheres and matched adherent cells were used to conduct quantitative real-time PCR for expression of Galectin-3. RT^2^ qPCR^TM^ primer assay for human LGALS3 and β-actin (SABiosciences, Qiagen, Valencia, CA) was used in combination with RT^2^ SYBER Green (SABiosciences). Quantitative PCR was performed using a Roto-Gene 3000 and Roto-Gene 6.0.14 software (Corbett Research, Qiagen). Comparative quantitation analysis was performed normalized to house-keeping genes with adherent cells set as reference.

### Statistical Analysis

Student's *t*-test was used to compare groups. One-way ANOVA with post-test for linear trend was used to compare multiple groups. For tumour growth assessment, a Gompertz fit of the data was performed (GraphPad Prism software, V 5.0). The level of statistical significance was set at 0.05.

## Results

### Tumourspheres derived from MCF-7 cells exhibit long-term expansion potential and self-renewal efficiency

**Figure 1:**
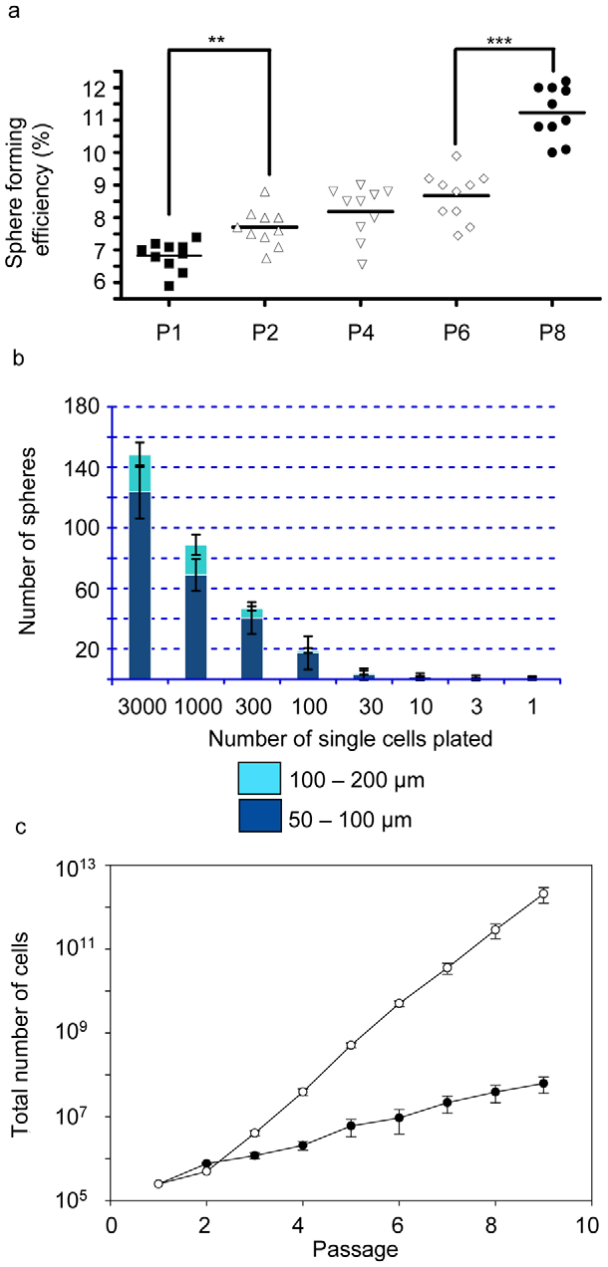
MCF-7 tumourspheres exhibit self-renewal efficiency and long term culture potential. Sphere forming efficiency progressively increases over serial passages (P). (a) Between P1 and P2 (*t*-test *p* = 0.0013, **), and between P6 and P8 (*t*-test *p*<0.0001, ***). (b) Sphere formation was dose dependent. Increasing the number of single cells plated in the sphere assay increased the total number of spheres formed. Sphere sizes range between 50 and 200 μm for day 5 spheres. (c) Growth curve showing total number of cells generated over 8 passages for matched adherent cells (open circles) compared to sphere-cultured cells (closed circles) demonstrating long-term culture capacity for spheres. Error bars represent standard error of the mean (SEM).

The MCF-7 cell line has been used to characterize tumour-initiating cells grown as non-adherent spheroids capable of sustained passage [7]. Albeit to a small degree, we found a statistically significant increase in sphere forming efficiency over serial passage for MCF-7 cells (Figure 1a), with sphere forming efficiency values ranging between 7 and 11%. These results are in line with those described by Cariati *et al*. [11] supporting the relevance of the sphere culture method for enriching cancer stem cells. Considering the discreet changes observed further biological parameters would need to be defined to further validate this interpretation. Self-renewing single cell precursors gave rise in a dose dependent manner to new tightly-packed multicellular spheres after a period of 5–7 days ranging in size between 50 and 200 μm (Figure 1b). Increasing numbers of single cells plated resulted in more spheroids and a greater total number of large spheroids (between 100 and 200 μm). Sphere-cultured cells were capable of long-term passage albeit at a lower rate of expansion than adherent cells (Figure 1c). The proliferation rate of spheres was consistent over eight passages. Previously results from our group have shown that MCF-7 spheres have a positive symmetric division rate of cancer stem cells [8] and that spheres are more tumourigenic than matched adherent cells [18]. Based on these results we compared the proteomes of early and late passage spheres and adherent cells.

### Proteomic analysis

Table S1 lists the 3542 proteins (sheet 1) identified that met the quantitation criteria (after filtering as described in the methods). The number of proteins identified compares favourably with prior studies using an offgel approach. Hubner *et al*., found 2892 proteins (from yeast extract) when using 12 well offgel in combination with an LTQ-Orbitrap mass spectrometer [24].

The effect of the different growth media and the microenvironment within the tumourspheres had a large effect on the expressed proteome of MCF-7 cells with 21% or 746 proteins (Table S1, sheet 2) showing statistically significant regulation in the *t*-test comparison (*p* value below 0.05) of P5 spheres and adherent cells. These results represent the first quantitative proteomic comparison of MCF-7 cells grown adherently and as spheres. The number of proteins showing statistically significant regulation in the comparison of P2 spheres and adherent cells was 462 (Table S1, sheet 3). This demonstrates that the proteomic techniques employed were capable of differentiating early and late passage spheres from adherent cells however, the comparison of P5 and P2 spheres yielded only 25 statistically significant regulated proteins (Table S1, sheet 4). Significantly these 25 proteins included CEACAM6, MUC5/MUC5AC, POLD2, IGFBP3, SPRR3, MCPIP1, BLMH; proteins associated with tumour progression/proliferation (SPRR3 [25]), dedifferentiation (POLD2 [26]), cell microenvironment/hypoxia (IGFBP3 [27]) and patient survival (MCPIP1 [28]); and represent those proteins most changed due to the differing properties of early and late passage tumourspheres.

Table 1 lists seven proteins that were present in greater amounts in P5 sphere-derived cells than adherent cells, and are expressed either on the plasma membrane or associated with plasma membrane proteins. Fold change from adherent conditions to P5 sphere conditions are presented with all showing significant regulation (all except LGALS1/galectin-1 were significant in the *t*-test comparison of passage 5 spheres and adherent cells). Several of these proteins demonstrated an apparent lack of protein expression in adherent conditions including: CEACAM6, CEACAM5, MUC1, MUC5AC, and MUC5B. MUC1 and galectin-3 were selected to validate the results of the proteomic approach in relation to differential abundances of specific surface marker proteins.

**Table 1:** Molecules of interest up-regulated in spheres compared to adherent cells.

Symbol	Name	ANOVA P-value^1^	Fold Change (Adherent to Passage5)	Location	Functional Description and/or Biological/Tumour Function
CEACAM6	carcinoembryonic antigen- related cell adhesion molecule 6	1.2E-05	5.0E08	Plasma Membrane	differentiation, cell adhesion, apoptosis, metastasis
CEACAM5	Carcinoembryonic antigen- related cell adhesion molecule 5	1.02E-02	2.17E08	Plasma Membrane	differentiation, cell adhesion, apoptosis
LGALS3	galectin-3	3.72E-04	2.71	Plasma Membrane, Cytoplasm	cell-cell and cell-matrix interactions, metastasis, anoikis resistance
LGALS1	galectin-1	9.71E-03	1.90	Plasma Membrane, Cytoplasm	migration, apoptosis, proliferation, angiogenesis
MUC1	Mucin 1, cell surface associated	4.53E-07	3.56E08	Plasma Membrane	glycoprotein, oncoprotein role as described in discussion
MUC5/MUC5AC	Mucin-5 subtype AC, tracheobronchial	3.66E-07	2.84E08	Extracellular Space	components of the mucus matrix forming family of mucins
MUC5/MUC5B	Mucin-5 subtype B, tracheobronchial	1.42E-07	4.62E08	Extracellular Space	components of the mucus matrix forming family of mucins

1) ANOVA p-values comparing adherent, passage2 spheres and passage 5 spheres.

### MUC1 expression is increased on spheres compared to adherent cells

**Figure 2:**
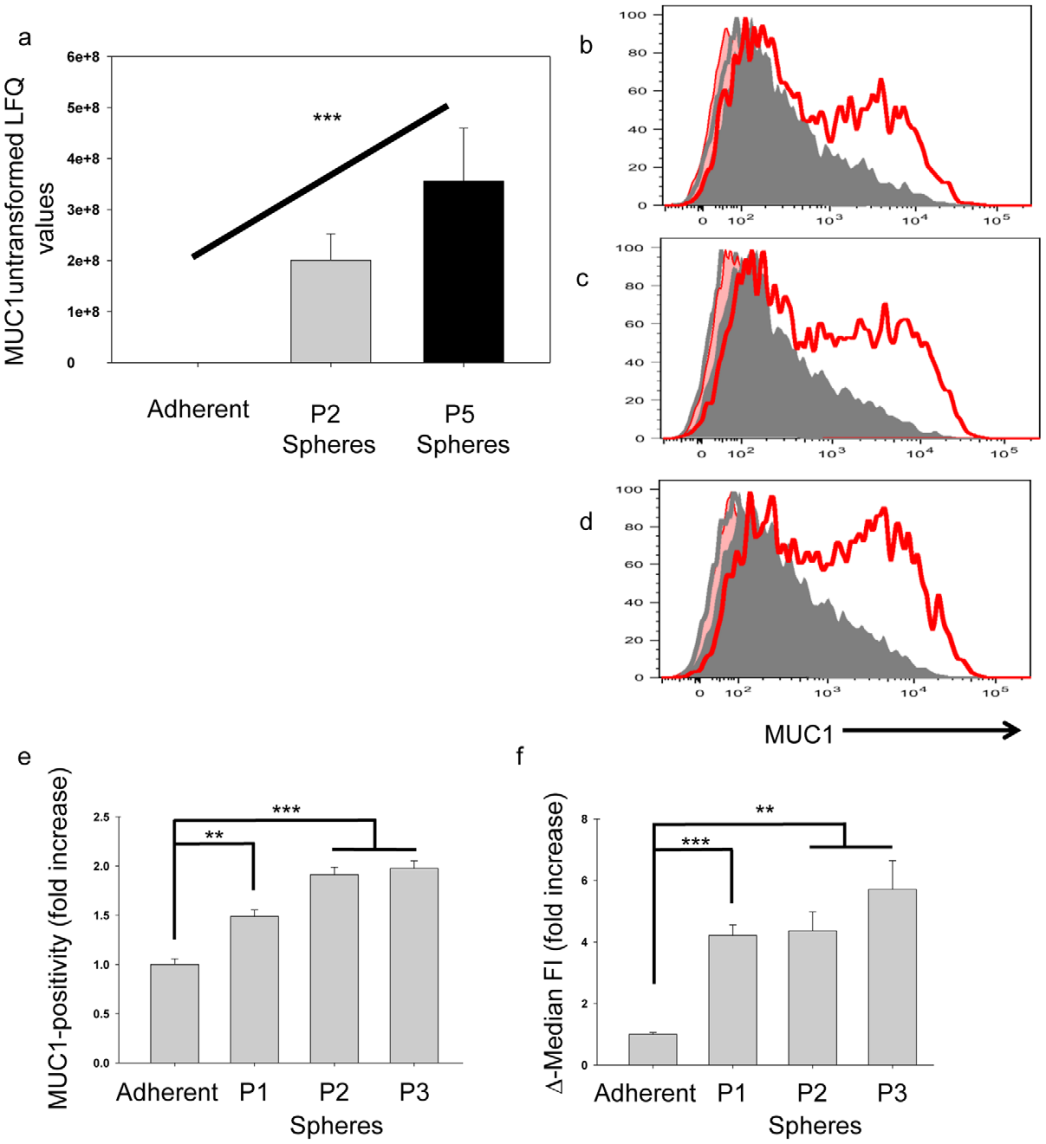
MUC1 expression on MCF-7 grown in matched adherent and sphere-promoting conditions. (a) Proteomics results (*y*-axis is untransformed label free quantitation (LFQ) values) indicate that the intensity of MUC1 expression is greater on sphere-cultured passage (P) 2 and P5 MCF-7 cells compared to adherent cells (line over columns), ANOVA test, *** *p*<0.001. (b–d) Surface expression of MUC1 was analyzed by flow cytometry on MCF-7 cells grown as matched adherent (shaded-grey) and sphere (red-line) cultures. The red shaded areas and grey lines represent the isotype controls for sphere and adherent cultures respectively. Sphere cultures were analyzed after 1 (b), 2 (c) and 3 (d) 6-day passages. Live-gated cells were analyzed for the frequency of MUC1-positivity (e) and Δ-median fluorescence intensity (FI) (f). Fold increase values for sphere cultures above the corresponding adherent culture are shown in bars representing the average and SEM of 3 biological repeats. Each biological repeat is the average of three technical repeats. Statistical significance of the change in MUC1 expression for each passage of spheres was calculated compared to adherent cells using a Student's *t*-test (*** *p*<0.001; ** *p*<0.01). Error bars represent SEM.

Proteomics indicated that MUC1 was exponentially more intensely expressed on P2 or P5 sphere-derived cells compared to adherent cells (Table 1, Figure 2a); ANOVA test *p*<0.001. MUC1 intensity was found to increase (not statistically by *t*-test) by 1.77 fold between passage 2 and 5 spheres (Figure 2a). To confirm these results, cell surface expression of MUC1 was further assessed by flow cytometry in matched adherent cells to passages 1, 2 and 3 spheres. Spheres were found to have an increased frequency of cells expressing MUC1 compared to adherents, and an increase in the Δ-median FI of MUC1 for spheres compared to adherent was also noted (Figure 2b–f).

### Galectin-3 and galectin-1 have increased expression within spheres compared to adherent cells

**Figure 3:**
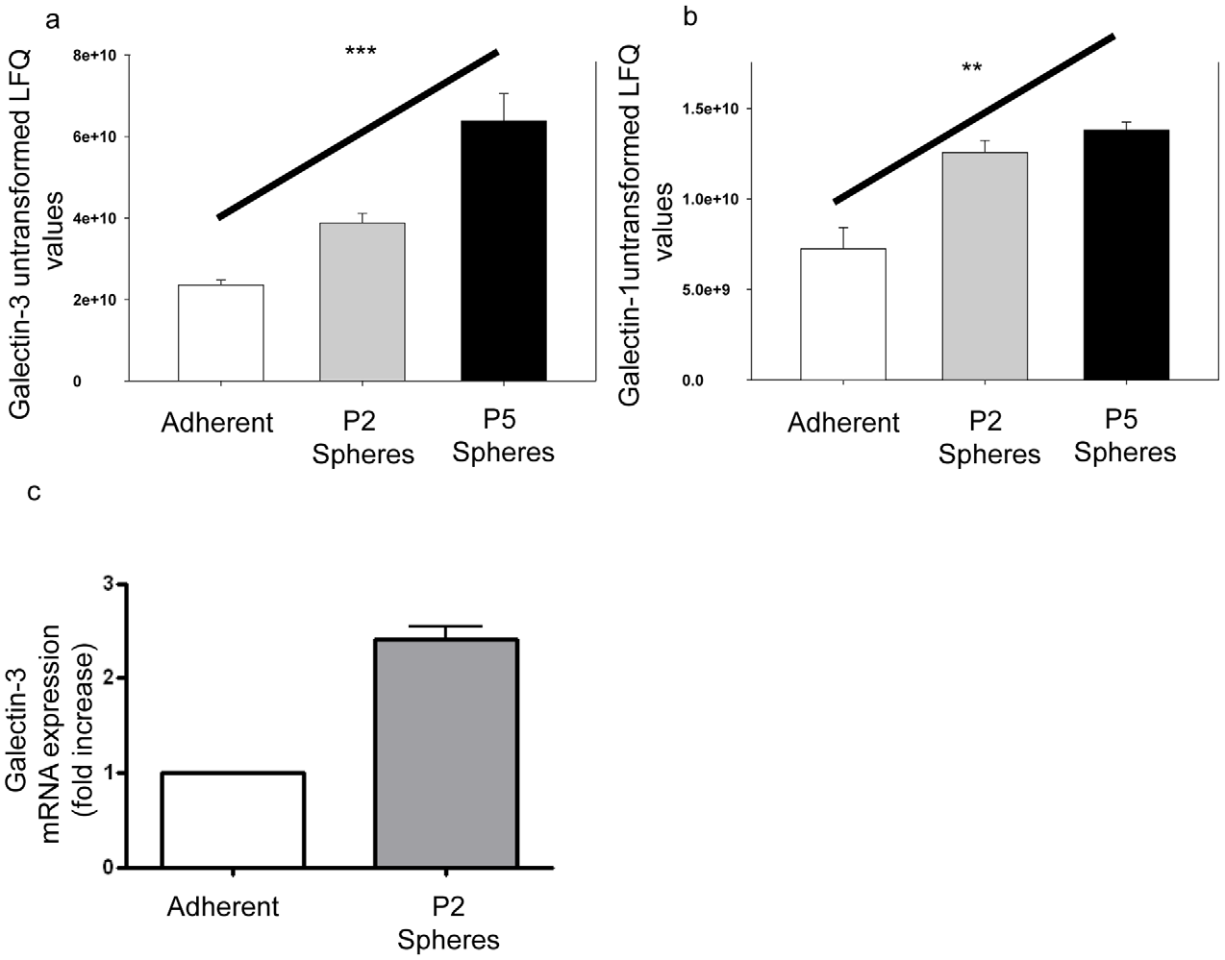
Galectin-3 expression on MCF-7 adherent cells and tumourspheres. Galectin-3 (a) and Galectin-1 (b) proteomics results (*y*-axis is untransformed label free quantitation (LFQ) values) indicate increased expression within spheres compared to adherent cells, (line over columns), ANOVA test, *** *p*<0.001; ** *p*<0.01. (c) Comparative quantitation of mRNA expression of Galectin-3 for adherent and P2 spheres. P2 spheres and adherent cells were analysed for expression of galectin-3 by quantitative RT-PCR. Results are displayed as fold increase relative to adherent cells and normalized to β-actin. Error bars represent SEM.

**Figure 4:**
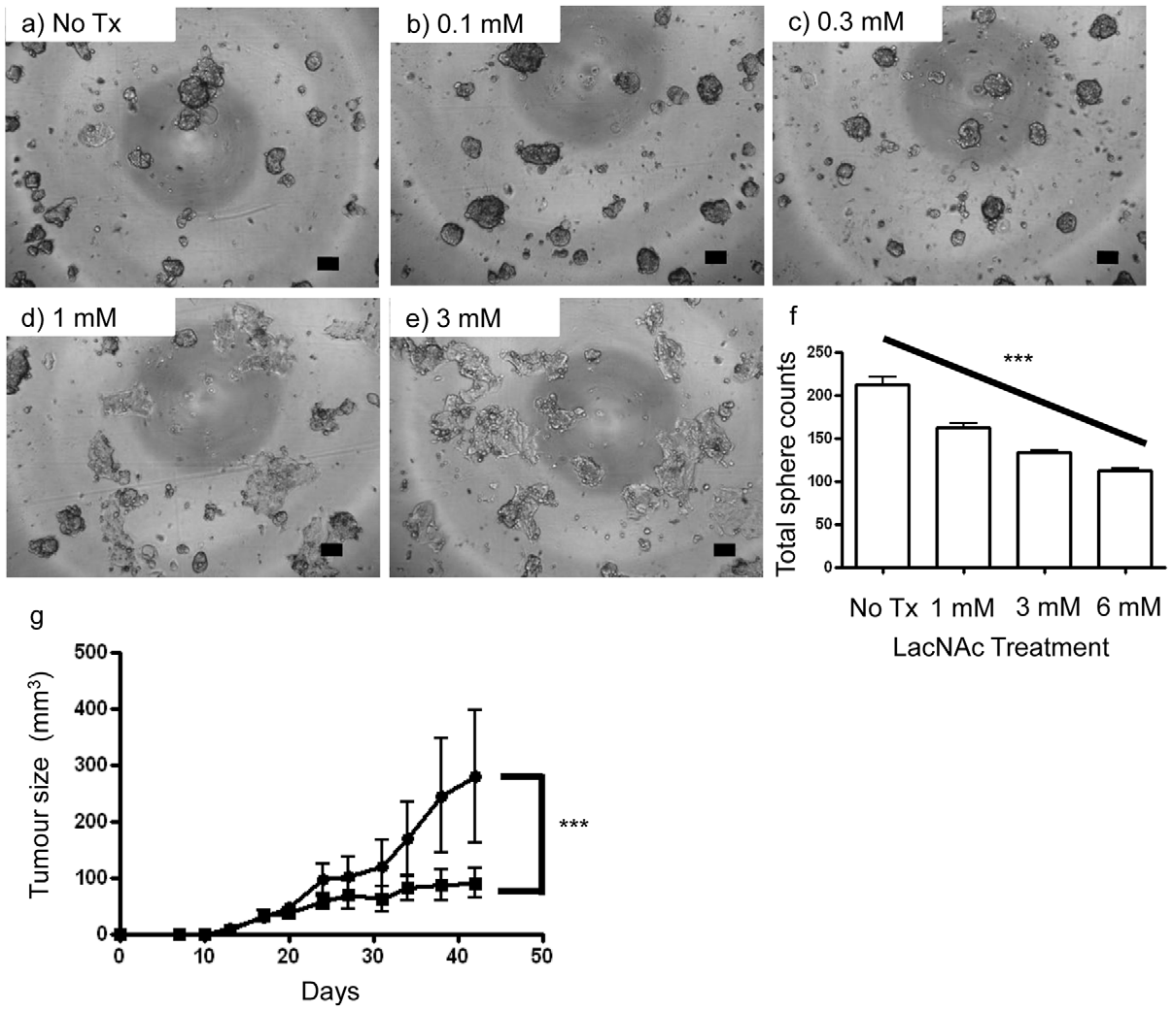
LacNAc effect on phenotype and tumourigenicity of MCF-7 tumourspheres. (a–e) Representative 4x photomicrographs of passage 2 spheres five days after culture in LacNAc containing sphere media. MCF-7 spheres cultured with LacNAc show a more differentiated morphology than those not cultured with LacNAc with most cells no longer forming spheres and attaching to the culture flask, as is seen under adherent conditions. (a) no treatment (No Tx), (b) 0.1 mM, (c) 0.3 mM, (d) 1 mM, and (e) 3 mM LacNAc. Bars represent 100 μm. LacNAc inhibits sphere forming frequency for MCF-7. Cells were cultured for 5 days in the sphere forming assay, 5000 cells per well. (f) A one-way ANOVA with a post-test for linear trend between number of spheres generated and amount of LacNac added demonstrated a significant decreasing trend (line over columns), *** *p*≤0.0001. Experiment repeated twice, representative results shown. Passage 2 MCF-7 spheres pre-treated with LacNAc have a slower tumour size enlargement then control cells (g). MCF-7 spheres were cultured in LacNAc at 3 mM for 7 days (closed squares) or left untreated (closed circles). Four immunocompromised mice (per group) were injected subcutaneously with 2×10^6^ cells. Mice were monitored for tumour growth twice weekly. A Gompertz fit model of the data indicated significant differences in tumourigenicity between treated and untreated groups (*** *p*<0.001). Error bars represent SEM.

Galectin-3 and galectin-1 were found to have increased protein expression within spheres compared to adherent cells using an ANOVA test to compare all three groups (Table 1, Figure 3a and 3b); galectin-3, *p*<0.001; galectin-1, *p*<0.01. P5 sphere-derived cells were found to increase expression of galectin-3 by 2.71 fold and galectin-1 by 1.9 fold compared to adherent cells. Further experiments demonstrated that at the mRNA level more Galectin-3 mRNA was expressed within P2 spheres than adherent cells utilizing quantitative RT-PCR (Figure 3c). Subsequent experiments tested the ability to target galectin-3 using the inhibitor N-acetyllactosamine (LacNAc) to demonstrate the utility of a proteome approach for identifying candidate BCSC markers. P2 spheres were grown for 5 days in the presence of 0, 0.1, 0.3, 1, or 3 mM LacNAc and morphology of cells/spheres and sphere forming efficiency was assessed (Figure 4a–4e). MCF-7 spheres cultured with LacNAc demonstrated an increase in cells adhering to the culture flask and a decrease in cells forming spheres. This decrease was dose dependent. MCF-7 cells demonstrated a statistically significant decrease in sphere formation with the addition of LacNAc (Figure 4f); ANOVA test *p*≤0.0001. Pre-treatment of cells with LacNAc *in vitro* affected tumour size *in vivo* in a pilot experiment (Figure 4g). A Gompertz fit of the data was supported by the replicates test, and indicated that tumour size was significantly different between the control group and cells that were pre-treated with LacNAc (*p*<0.001).

## Discussion

In this study we have characterised the proteome of MCF-7 cells grown as adherent monolayers, and as tumourspheres at two different times of culture. Comparing Passage 2 and Passage 5 tumourspheres the proteomic analysis identified 25 proteins significantly changing in abundance even though sphere forming efficiency only increased by about 13%. This demonstrates that the techniques employed can identify changes in cells even when the overall cell population is not drastically changed. The comparison of adherently grown monolayers and tumourspheres identified many more proteins (746), with a lot of the change a result of the changed environment and growth medium, however many proteins previously associated with a cancer stem cell phenotype were observed to change in abundance.

Cancer stem cells are identified by their ability to grow as self-renewing tumourspheres *in vitro* and to initiate tumours *in vivo* [7], [13]. We have previously shown that MCF-7 sphere cultured cells are more tumourigenic in immunocompromised mice than parental adherent cells [18] and that MCF-7 spheres have a symmetric division frequency indicating stem cell activity [8]. In this study, we confirm previous observations [7], [11] that sphere-cultured MCF-7 cells are capable of sustained growth as spheres over serial passage and that sphere formation is enriched on later passage spheres compared to earlier passage spheres. We also demonstrate that the number of spheres formed is dependent on the number of single cells cultured and that spheres can form from few cells (less than 30 cells cultured). Thus, the sphere assay is a useful assay for investigating cancer stem cells in comparison to monolayer adherent culture. For these reasons we explored the proteome of spheres *vs*. adherent cultured MCF-7 cells. Recently a proteomic analysis has been conducted for MCF-7 monolayer cells in comparison to another commonly used breast cancer cell line T-47D [29]. We believe this to be the first time that the proteome of multiple passages of MCF-7 spheres have been compared to monolayer cells.

Proteomic analysis is a leading research methodology for understanding tissue protein expression. One theoretical drawback of proteomic research is that identification of plasma membrane proteins is often difficult to perform due to several reasons: (i) plasma membrane proteins are often more hydrophobic and therefore less soluble than cytosolic proteins; (ii) they often have significant post-translational modifications (such as glycosylation, phosphorylation and lipid moieties) making identification difficult; and (iii) they are not as relatively abundant as other proteins (reviewed in [30]). Despite these shortcomings in identifying cell surface markers, proteomics still offers a powerful high-throughput technique to collect a large dataset of proteins to further investigate and test hypotheses with, and in the present study we were able to identify proteins of interest that did have plasma membrane cellular locations. We hypothesized that the proteome of sphere-derived MCF-7 cells would identify candidate proteins involved in tumoursphere formation/function and cancer stem cell activity compared to the proteome of monolayer cells.

Interestingly, a similar study has recently been published which used a proteomic approach to define markers of the BCSC phenotype [31]. That study identified a different set of protein markers to those identified in the present study. However, the two studies used different cell lines, MCF-7 cells in our study and primary mouse cell lines in the other study. Furthermore, quite different proteomic approaches were used at a technical level. These differences may account for the finding of different markers for the BCSC phenotype in the two studies. Together, these two studies can be considered complementary as they provide a larger range of options for studying the BCSC phenotype. The previous study highlighted the ferritin heavy chain as a major BCSC marker. This protein was observed to be regulated upon superficial analysis of data from our study, however, the 2–3 fold regulation apparent for ferritin heavy chain in our data was not statistically significant due to inherent biological variability that was evident when all of the independent biological replicates were taken into account. Indeed, due to the well of information made available with techniques such as the ones described here, biological variability is an important consideration that needs to be taken into account when assessing results of proteomics research.

Several proteins were found to be more highly expressed within spheres compared to adherent cells. We initially focused our attention on proteins that were differentially expressed between our late passage (passage 5) spheres compared to adherent cells. Amongst these, there were found to be several proteins associated with the plasma membrane (CEACAM5/CEACAM6, MUC1, galectin-1, and galectin-3 amongst others). Plasma membrane proteins were targeted because they can readily be used for further validation and as tools for enriching and/or targeting BCSC. We found exponentially increased expression for CEACAM5 and CEACAM6 proteins for late passage spheres compared to adherent cells, significantly the increase in CEACAM6 occurred between early and late sphere passage. High expression of CEACAM5 and CEACAM6 has been associated with a variety of malignancies including breast cancer [32], [33], [34]. CEACAM5 and CEACAM6 have described roles associated with; tumour cell chemosensitivity; (ii) cell adhesion; (iii) invasion; (iv) metastasis; and (v), importantly to the field of cancer stem cells, these molecules have been described to inhibit anoikis [35], [36], [37]. One characteristic of tumoursphere culture conditions that is different to adherent culture is that during passaging of spheres a majority of cells die (through apoptosis/anoikis) early on and the mitogen-responsive anoikis resistant cells (putative cancer stem cells) proliferate and form new spheres [8]. Increased expression of these two proteins within spheres compared to adherent cells could be a result of spheres being enriched for anoikis-resistant cancer stem cells. Additionally, antibodies targeting these molecules have been shown to inhibit adhesion, invasion, and metastasis *in vitro* and *in vivo* [35], demonstrating the ability to direct therapy against cells expressing these targets. Therefore, CEACAM5 and CEACAM6 may be promising targets for cancer immunotherapy including, perhaps, immunotherapy aimed at targeting breast cancer stem cells and metastatic disease.

Results from proteomics demonstrated an exponential increase in MUC1 expression within spheres compared to adherent cells. These results were confirmed by flow cytometry, indicating increased expression within spheres compared to adherent cells. MUC1 is a Type I membrane glycoprotein of the mucin family normally expressed at the apical border of healthy epithelial cells [38], [39]. In cancerous tissue, MUC1 protein expression is aberrantly expressed on multiple cell surfaces on as much as 75% of human solid tumours [40] and greater than 90% of human breast cancer and subsequent metastases [41]. Increasing evidence has demonstrated a role for MUC1 as an oncoprotein, including demonstrations of a role in promoting increased cell growth rate [42], in anti-apoptosis [40], and in promoting anchorage independent cell growth [43]. Additionally, a low molecular weight MUC1 cleavage product, MUC1*, that remains membrane bound has recently been shown to be a determinant of trastuzumab resistance in HER2^+^ breast cancer [44] and functions as a growth factor receptor [45], [46]. MUC1 has been proposed as a target for immunotherapy of breast cancer with some clinical trials exploring various MUC1-based vaccines having been conducted [47], [48], [49]. Tumour-associated alterations of MUC1 such as hypoglycosylation, increased sialyation, and altered carbohydrate core-type expression are responsible for the antigenicity of MUC1 and hence its suitability as a target for immunotherapy [38], [50], [51]. MUC1 expression has recently been reported to be expressed on the side population cells from MCF-7 mammosphere cultures demonstrating for the first time that MUC1 is not only expressed on mature breast cancer cells but also on cells that have some of the functional characteristics of cancer stem/progenitor cells [15]. Immunotherapy approaches that target breast cancer stem/progenitor cells has the potential to eliminate minimal residual disease and may lead to more meaningful clinical remissions [3]. Our results indicate that MUC1 expression is enhanced on cells with a stem-cell like phenotype, and that this expression is consistent over multiple passages of these cells as spheres. Further characterisation of MUC1 expression on stem/progenitor cells will be instrumental for the future application of MUC1-based tumour vaccines.

Galectin-1 and Galectin-3 were found to have increased expression on P5 sphere-derived cells compared to adherent cells (approximately 1.9 and 2.71 fold increase respectively). Galectin-1 and -3 are members of the lectin family, selectively bind β-galoctoside residues and consist of a carbohydrate recognition domain, a collagen-like domain, and a NH2-terminal domain. Galectin-1 has a described role in mediating cell adhesion and migration [52], apoptosis [53], proliferation [54], and in facilitating tumour angiogenesis [55]. As such, the increase in expression for this protein from adherent cells to P5 spheres suggests that spheres are enriched with cells that play a role in metastasis and survival to therapy – putative cancer stem cells. A further candidate cancer stem cell molecule identified in our model was galectin-3. Galectin-3 is located in cytoplasmic, nuclear and extracellular sites [56]. Galectin-3 has several described associations and roles related to cancer, in particular in relation to cell-cell and cell-matrix interactions, metastasis, angiogenesis, tumour progression and resistance to apoptosis/anoikis (reviewed in [57], [58]). Galectin-3 enhances metastasis through promoting tumour cell adhesion [59], [60], invasiveness [61], and inducing tumour cell proliferation and angiogenesis [62]. Galectin-3 also antagonises tumour cell apoptosis and anoikis [63], [64], [65]. Our finding that galectin-3 is enhanced in spheres compared to adherent cells may be a reflection of the anoikis-resistant characteristic of cells in sphere culture.

In relation to breast cancer, galectin-3 has a described role in enhancing metastatic disease through resistance to the products of inducible nitric oxide synthase and through its bcl-2-like anti-apoptotic properties [66]. One study investigating alterations in galectin-3 expression and distribution within tumour cells in a mouse xenograft model has demonstrated that upregulated galectin-3 is correlated with breast cancer progression [67]. Hence, Galectin-3 is a promising target for cancer therapy. One molecule used as a competitive inhibitor of natural ligands for galectin-3 is the polysaccharide modified citrus pectin (MCP). *In vitro* MCP has been shown to inhibit; (i) aggregation of tumour cells; (ii) angiogenesis; and (iii) tumour cell adhesion to endothelial cells [62], [68], [69]. *In vivo* MCP has been shown to inhibit metastasis for intravenous and oral administration in animal models of metastatic B16-F1 melanoma [68], prostate adenocarcinoma [69] and human breast carcinoma cells [70]. Galectin-3 null mice have been shown to be relatively healthy [71], suggesting that inhibition of galectin-3 might be therapeutically valuable while sparing patients from severe side effects. We have used LacNAc as an inhibitor to investigate the role of galectin-3 in tumoursphere formation *in vitro*, demonstrating that LacNAc can alter tumoursphere formation. Unfortunately, natural saccharide ligands of galectin-3 (such as LacNAc) typically display a low micromolar affinity for galectin-3 and as such many researchers are investigating modifying LacNAc for higher affinity with galectin-3 in particular for use within *in vivo* models [71], [72]. We have demonstrated that the use of LacNAc prevents the formation of tumourspheres in a dose dependent manner for MCF-7 breast cancer cells. MCF-7 single cells treated with LacNAc and placed into sphere culture resulted in fewer recognizable spheres formed as the cells were more likely to adhere to the tissue culture flask than to each other. This could be due to the ligand blocking the homotypic adhesion of cells together in such a way as cells are more likely to stick to the tissue culture flask than to each other. Another way that sphere formation could be blocked is by LacNAc preventing the function of long-term proliferating cells (putative BCSCs) to self-renew and form spheres.

These experiments highlight the ability to use proteomics to identify candidate proteins involved in tumoursphere formation/function. It is important to note that further validation of these candidate proteins will need to take into account isolation of individual cell populations within tumourspheres to assess their stem cell-like phenotype in order to conclusively state that they are associated with cancer stem cells. We made use of the tumoursphere assay as a read-out for self-renewal efficiency of cells within tumourspheres. This assay is a good *in vitro* approach to investigating cells with stem cell-like functions. However, future experiments will need to address the tumour-initiating properties of cells identified/isolated based on candidate cancer stem cell-like proteins in *in vivo* models.

In conclusion, the present study shows a proof of principle for using proteomics to investigate differences between protein expression by early and late tumourspheres or by tumourspheres and adherent cells. This approach can be used to identify candidate cancer stem cell associated proteins for further investigations and for therapy intervention. We have identified a number of proteins enriched within spheres relating to (breast) cancer and stem cell functions. Further investigations into one of these proteins, galectin-3, demonstrates that targeting a marker enriched on breast cancer stem cells affects self-renewal of cancer stem cells and tumourigenicity *in vivo*. This study demonstrates that tumoursphere cells and adherent cells possess markedly different proteomes. Importantly, data generated could lead to the identification of novel markers that could be used to improve pathological diagnoses and/or as targets for future breast cancer intervention.

## Supporting Information

Table S1 Total list of proteins identified using the MaxQuant method. List of 3542 proteins identified and analysed for differential expression of proteins amongst the three groups via ANOVA and *t*-test from adherent cells to P2 spheres to P5 spheres. Intensity of counts is shown for the three biological replicates within each group. ANOVA and *t*-test *p* values are as indicated.
(XLSX)
